# Bayesian Thurstonian IRT Modeling: Logical Dependencies as an Accurate Reflection of Thurstone’s Law of Comparative Judgment

**DOI:** 10.1177/00131644251335586

**Published:** 2025-05-30

**Authors:** Hannah Heister, Philipp Doebler, Susanne Frick

**Affiliations:** 1 University of Groningen, Groningen, Netherlands; 2 Technische Universität Dortmund, Dortmund, Germany

**Keywords:** Thurstonian item response theory, Markov Chain Monte Carlo, Bayesian item response theory

## Abstract

Thurstonian item response theory (Thurstonian IRT) is a well-established approach to latent trait estimation with forced choice data of arbitrary block lengths. In the forced choice format, test takers rank statements within each block. This rank is coded with binary variables. Since each rank is awarded exactly once per block, stochastic dependencies arise, for example, when options A and B have ranks 1 and 3, C must have rank 2 in a block of length 3. Although the original implementation of the Thurstonian IRT model can recover parameters well, it is not completely true to the mathematical model and Thurstone’s law of comparative judgment, as impossible binary answer patterns have a positive probability. We refer to this problem as stochastic dependencies and it is due to unconstrained item intercepts. In addition, there are redundant binary comparisons resulting in what we call logical dependencies, for example, if within a block A<B and B<C , then A<C must follow and a binary variable for A<C is not needed. Since current Markov Chain Monte Carlo approaches to Bayesian computation are flexible and at the same time promise correct small sample inference, we investigate an alternative Bayesian implementation of the Thurstonian IRT model considering both stochastic and logical dependencies. We show analytically that the same parameters maximize the posterior likelihood, regardless of the presence or absence of redundant binary comparisons. A comparative simulation reveals a large reduction in computational effort for the alternative implementation, which is due to respecting both dependencies. Therefore, this investigation suggests that when fitting the Thurstonian IRT model, all dependencies should be considered.

## Introduction

Personality questionnaires are ubiquitous in most areas of psychological assessment and education. Constructs like personality and motivation can explain variance in achievement beyond ability. However, an accurate scoring of responses to personality questionnaires is necessary for decisions based on them to be reliable and fair. The multidimensional forced-choice (MFC) format has become popular as a response format in personality questionnaires. In the MFC format, participants have to rank order items measuring different attributes. The MFC format avoids response biases such as, for example, halo effects (Brown et al., 2017) or extreme response style (Brown & Maydeu-Olivares, 2018). Furthermore, it reduces faking compared to rating scales (Cao & Drasgow, 2019; Wetzel et al., 2021).

The scoring of MFC data according to classical test theory results in ipsative scores. Ipsative scoring distorts correlation-based analyses (Clemans, 1966) and the scores should not be compared between persons (Closs, 1996; Johnson et al., 1988). Normative scoring of MFC data has become possible with advances in the computation of item response theory (IRT) models. Brown (2016) gives an overview of IRT models for forced-choice response formats. The most popular IRT model for MFC data is the Thurstonian IRT (T-IRT) model (Brown & Maydeu-Olivares, 2011). Thurstonian IRT scoring of MFC data results in normative scores (Brown & Maydeu-Olivares, 2013; Frick et al., 2023). Moreover, the Thurstonian IRT model is the most widely applicable IRT model for MFC data since it can accommodate various response formats and instructions (Brown & Maydeu-Olivares, 2018). To estimate the Thurstonian IRT model, the rankings are re-coded into pairwise item comparisons. Each possible combination of two items is considered once. Exemplary, this is illustrated for a block of length 3 with the realized ranking (2,3,1) :

$$\begin{array}{ccccccc} \mathrm{Block} & & \mathrm{Ranking}\;\mathrm{of}\;a & & \mathrm{Pairwise} & & \mathrm{Binary}\\ & & \mathrm{person} & & \mathrm{comparisons} & & \mathrm{outcomes}\\ \begin{array}{c} \mathrm{Item}\;A\\ \mathrm{Item}\;B\\ \mathrm{Item}\;C \end{array} & \to & \left(\begin{array}{c} 2\\ 3\\ 1 \end{array}\right) & \to & \begin{array}{c} \{A,B\}\to 2<3\\ \{A,C\}\to 2>1\\ \{B,C\}\to 3>1 \end{array} & \to & \left(\begin{array}{c} 1\\ 0\\ 0 \end{array}\right) \end{array}$$

In the original implementation (Brown & Maydeu-Olivares, 2011, 2012), the intercepts for the pairwise comparisons are unconstrained, that is, a set of item intercepts that comply with them does not have to exist. However, since the data are indeed item rankings, they imply *stochastic* dependencies that this model specification does not account for, as we will show in the following.

In addition, in the original implementation, the person parameters were estimated with maximum likelihood or maximum a posteriori estimation. This person parameter estimation is based on the product of dependent normal distributions (for block sizes >2 ) and therefore overestimates the precision and underestimates the standard errors (Brown & Maydeu-Olivares, 2011; Frick et al., 2023; Yousfi, 2020). The same applies to the Bayesian implementation in the R package thurstonianIRT (Bürkner et al., 2019). Based on the observation that ranking data can be correctly expressed using a multivariate distribution and considering only independent pairwise comparisons (Maydeu-Olivares, 1999), Yousfi (2020) proposed an alternative method for person parameter estimation that yields correct standard errors. We will term the dependencies that arise between redundant pairwise comparisons *logical* dependencies in the following.

This research aims to investigate the effect of considering logical dependencies, both analytically (for block size 3) and in a simulation (for block sizes 3 and 4). We show analytically that logical dependencies do not play a role as long as stochastic dependencies are considered. We propose a new Bayesian implementation of the Thurstonian IRT model using a multivariate distribution. In the following, we will first present the Thurstonian IRT model, then explicate our definitions of stochastic and logical dependencies and give an intuition of the related proof. The full proof can be found in the Appendix. Afterward, we will present a simulation that compares three implementations: considering both types of dependencies, neglecting logical ones, and the original that neglects both types of dependencies. We will end with a discussion on the implications of our investigation for item and person parameter estimation in practice.

## The Thurstonian IRT Model

The Thurstonian IRT model is based on the law of comparative judgment (Thurstone, 1927), which states that test takers compare statements pairwise when ranking multiple options. This justifies that a person’s ranking can be viewed as a series of binary item comparisons. It is assumed that the rank of each statement within a block is determined by the test takers latent utility toward that statement. Next to statement-specific characteristics, a test taker’s utility toward a statement is influenced by latent traits θ , common factors ideally corresponding directly to psychological constructs. For test taker j , the utility $t_{i}^{\;j}$ toward statement i is expressed as the linear function:

(1) $$t_{i}^{\;j}=\mu_{i}+\lambda_{i}\theta_{j}+\varepsilon_{\mathrm{ji}},$$

where $\mu_{i}$ denotes the latent utility mean, $\lambda_{i}$ the vector of factor loadings of the statement i on the test taker’s latent traits $\theta_{j}\sim^{\mathrm{iid}}N\left(0,\Phi \right)$ , and ε the random error. The errors in each statement’s utility are assumed to be independent $\epsilon_{j}\sim^{\mathrm{iid}}N\left(0,\Psi \right)$ , while the latent traits can correlate. In the following, we assume that each statement only measures one trait (simple structure); therefore, $\lambda_{i}$ has only one non-zero entry, denoted by $\lambda_{i}^{\left(1\right)}$ . When test takers rank statements, the statements are ranked in order of their utility. The higher the utility, the higher the statement is ranked. The binary outcome for two statements i and l can therefore be expressed as:

(2) $$\begin{array}{cc} y_{\mathrm{il}}^{\;j}=\left\{ \begin{array}{c} 1\;\mathrm{if}\;t_{i}^{\;j}-t_{l}^{\;j}:=y_{\mathrm{il}}^{\;j*}\geq 0,\\ 0\;\mathrm{else},\;i.e.,\;t_{i}^{\;j}-t_{l}^{\;j}=y_{\mathrm{il}}^{\;j*}<0. \end{array}\right. & \end{array}$$

Note that there is no further error term as the ranking is considered to be transitive. The probability of test taker j preferring statement i over statement l can be calculated from the model parameters with:

(3) $$P\left(y_{\mathrm{il}}^{\;j}=1|\theta_{j},\gamma ,\Lambda ,\Psi \right)=\Phi \left(\frac{-\gamma_{\mathrm{il}}+\lambda_{i}\theta_{j}-\lambda_{l}\theta_{j}}{\sqrt{\psi_{\mathrm{ii}}+\psi_{\mathrm{ll}}}}\right),$$

where $\psi_{\mathrm{ii}}$ is the i -th diagonal entry of Ψ .

To represent the full response pattern in a block of length $n_{b}$ , all $n_{b}\cdot \left(n_{b}-1\right)/2$ binary comparisons are considered, although some could be redundant by transitivity. Each statement is compared with its subsequent statements in the indexing of statements. The compared statements are coded in a design matrix A of the comparisons that determines which utility differences are computed. Each row of A relates to one of the $n_{b}\cdot \left(n_{b}-1\right)/2$ binary comparisons while each column corresponds to one of the $n_{b}$ statements. For a single block of length $n_{b}=3$ , the design matrix A is therefore defined as:

(4) $$A=\left(\begin{array}{ccc} 1 & -1 & 0\\ 1 & 0 & -1\\ 0 & 1 & -1 \end{array}\right).$$

When modeling multiple K blocks instead of just one, A becomes a block diagonal matrix with dimensions $A\in R^{K\cdot \left(n_{b}\cdot \left(n_{b}-1\right)/2\right)\times K\cdot n_{b}}$ . The response model of the full questionnaire for test taker j expands to:

(5) $$y^{\;j*}=At^{\;j}=A\left(\mu +\Lambda \theta_{j}+\epsilon_{j}\right)=\underset{=:-\gamma }{\underset{︸}{A\mu }}+\underset{=:\tilde{\Lambda }\theta_{j}}{\underset{︸}{A\Lambda \theta_{j}}}+\underset{=:{\tilde{\epsilon }}_{j}}{\underset{︸}{A\epsilon_{j}}},$$

where Λ is the matrix of the factor loading with row vectors $\lambda_{i}$ . The constraint −γ=Aμ is not imposed in the original model (Brown & Maydeu-Olivares, 2011), and an unrestricted −γ is estimated. We will discuss the implications of this simplification in the next section. The vector of utility differences $y^{\;j*}$ is multivariate normal,

(6) $$y^{\;j*}|\theta_{j},\gamma ,\Lambda ,\Psi \sim N(-\gamma +A\Lambda \theta_{j},A\Psi A^{T}),$$

and since the covariance matrix $A\Psi A^{T}$ is not diagonal, dependencies between statement comparisons exist. In fact, we will discuss below that since A does not have full rank, the normal distribution is degenerate with a singular covariance matrix $A\Psi A^{T}$ .

## Dependencies in the Thurstonian IRT Model

The Thurstonian IRT model utilizes that each ranking can be unequivocally decoded into a pattern of binary comparisons. The other way around, this does not hold true: By transitivity, out of all $2^{n_{b}}$ binary patterns, only $n_{b}!$ can result from a ranking. This leads to dependencies in the binary pattern for a block length larger than two. Vividly, this can be illustrated by the pattern (1,0,0) , which was already considered in the introduction. When we know about a block consisting of statements A,B,C , that A is ranked above B and that A is ranked below C we can directly follow that B is ranked below C . Ranking C below B would be illogical. For all binary patterns, it holds true that only $\left(n_{b}-1\right)$ well-chosen comparisons are sufficient to derive the full pattern. The original Thurstonian IRT only partially considers those dependencies. We proceed by disentangling what we call stochastic and logical dependencies.

### Stochastic Dependencies

In the T-IRT model, intercepts γ are not restricted, for example, maybe counter to what one might expect, $\gamma_{\mathrm{AB}}-\gamma_{\mathrm{AC}}=\gamma_{\mathrm{BC}}$ must not hold true. Thus, the model does not fully account for dependencies on the item parameters, making the realization of illogical, intransitive patterns of binary comparisons theoretically possible. This can be illustrated by creating the intransitive pattern (1,0,1) for test taker j . For this, $y_{\mathrm{AB}}^{\;j*}>0,y_{\mathrm{AC}}^{\;j*}<0$ and $y_{\mathrm{BC}}^{\;j*}>0$ must hold. When considering the sum of $y_{\mathrm{AB}}^{\;j*}$ and $y_{\mathrm{BC}}^{\;j*}$ , this results in:

(7) $$\left(\underset{:=\gamma_{\mathrm{AB}}^{\;j*}>0}{\underset{︸}{-\gamma_{\mathrm{AB}}+\lambda_{A}\theta_{j}-\lambda_{B}\theta_{j}+\varepsilon_{\mathrm{jA}}-\varepsilon_{\mathrm{jB}}}}\right)+\left(\underset{:=\gamma_{\mathrm{BC}}^{\;j*}>0}{\underset{︸}{-\gamma_{\mathrm{BC}}+\lambda_{B}\theta_{j}-\lambda_{C}\theta +\varepsilon_{\mathrm{jB}}-\varepsilon_{\mathrm{jC}}}}\right)$$

(8) $$=-\gamma_{\mathrm{AB}}-\gamma_{\mathrm{BC}}+\lambda_{A}\theta_{j}-\lambda_{C}\theta_{j}+\varepsilon_{\mathrm{jA}}-\varepsilon_{\mathrm{jC}}>0.$$

The comparison with $y_{\mathrm{AC}}^{\;j*}=\left(-\gamma_{\mathrm{AC}}+\lambda_{A}\theta_{j}-\lambda_{C}\theta_{j}+\varepsilon_{\mathrm{jA}}-\varepsilon_{\mathrm{jC}}\right)<0$ shows that the illogical pattern can only be realized if:

(9) $$-\gamma_{\mathrm{AB}}-\gamma_{\mathrm{BC}}>\lambda_{C}\theta_{j}-\lambda_{A}\theta_{j}+\varepsilon_{\mathrm{jC}}-\varepsilon_{\mathrm{jA}}>-\gamma_{\mathrm{AC}}$$

holds true. While this is possible, if $\gamma_{\mathrm{il}}$ are independently estimated, this is not possible, if the constraint γ=−Aμ is imposed. Imposing the constraint ensures that $-\gamma_{\mathrm{AB}}-\gamma_{\mathrm{BC}}=-\gamma_{\mathrm{AC}}$ holds true. To avoid illogical patterns and to take the stochastic dependencies fully into account, γ has to be constrained. Only the model with constrained intercepts completely fulfills Thurstone’s law.

### Logical Dependencies

Another concern is the fact that the information between considered binary comparisons is partially redundant which results in logical dependencies. To circumvent these dependencies, one can take only neighboring ranking comparisons into account, which results in $\left(n_{b}-1\right)$ independent comparisons per block (Maydeu-Olivares, 1999; Yousfi, 2020). This is sufficient to derive the full pattern, as long as stochastic dependencies are considered. The corresponding proof for block length 3 is in the Appendix. For larger block lengths, an analogous proof can be developed.

For an intuitive argument, assume a,b , and c correspond to the true test taker’s utility differences. Then, the probability density of realizing the utility difference corresponding to the full binary pattern is equal to the probability density of realizing the utility difference corresponding to the reduced binary pattern times the Dirac measure $\delta_{y_{\mathrm{AC}}^{\;j*}}\left(a+c\right)$ :

(10) $$p\left(y_{\mathrm{AB}}^{\;j*}=a,y_{\mathrm{AC}}^{\;j*}=b,y_{\mathrm{BC}}^{\;j*}=c\right)$$

(11) $$\begin{array}{c} =p\left(y_{\mathrm{AB}}^{\;j*}=a,y_{\mathrm{BC}}^{\;j*}=c\right)\cdot \underset{︸}{p\left(y_{\mathrm{AC}}^{\;j*}=b|y_{\mathrm{AB}}^{\;j*}=a,y_{\mathrm{BC}}^{\;j*}=c\right)}\\ \begin{array}{c} =p\left(t_{A}^{\;j}-t_{C}^{\;j}=b|t_{A}^{\;j}-t_{B}^{\;j}=a,t_{B}^{\;j}-t_{C}^{\;j}=c\right)\\ =p\left(t_{A}^{\;j}-t_{C}^{\;j}=b|t_{A}^{\;j}-t_{C}^{\;j}=a+c\right) \end{array} \end{array}$$

(12) $$=p\left(y_{\mathrm{AB}}^{\;j*}=a,y_{\mathrm{BC}}^{\;j*}=c\right)\cdot \left\{ \begin{array}{c} 1,\;\mathrm{if}\;y_{\mathrm{AC}}^{\;j*}=a+c\\ 0,\;\mathrm{if}\;y_{\mathrm{AC}}^{\;j*}\neq a+c \end{array}\right.$$

(13) $$=p\left(y_{\mathrm{AB}}^{\;j*}=a,y_{\mathrm{BC}}^{\;j*}=c\right)\cdot \delta_{y_{\mathrm{AC}}^{\;j*}}\left(a+c\right).$$

If stochastic dependencies are considered, the Dirac measure is always one and the density of the subset of utility differences is identical to the density of all utility differences. Hence, the density of $y^{*}$ can be defined as the density of a subset of utility differences. If stochastic dependencies are not considered, impossible patterns can have a non-zero probability. For block length greater than two, not every subset of $\left(n_{b}-1\right)$ comparisons is sufficient. It is the subset of neighboring comparisons that is sufficient for every block length.

More so, this subset is sufficient to derive the full answer pattern. Why would a different subset of size $n_{b}-1$ not suffice? This can be illustrated by the statements A,B,C , where A>B and A>C . We cannot infer whether B or C has a higher utility. In other words, the most informative comparisons are needed to recover the answer pattern—the comparisons between statements with neighboring ranks. The closer the ranks, the lower the uncertainty about the utility differences and hence the latent variables. This becomes clear from an IRT perspective: When the probability of preferring A over B is 0.5 , the binomial likelihood of the binary comparison implies that the Fisher information of the latent variable is the largest. An optimal probability of .5 practically means that data on utilities close to each other are more informative. Note that the best and worst-ranked statements hold less information about the absolute position of the corresponding $\theta_{j}$ than other statements. Which statements have a neighboring rank depends on the test taker’s answer pattern. Hence, in the following, the design matrix A becomes a person-specific matrix $A_{j}$ which is only equal for persons with the exact same or the exact opposite ranking.

Conditional on other parameters, the answer pattern probabilities are products of the probabilities of single binary comparisons. The proposed estimation process directly uses the multivariate structure of the binary comparisons, accounting for logical dependencies in the process. This leads to a more economical use of data. While this could make the estimation process more efficient, it does not affect the estimated model, as shown in the Appendix.

**Figure 1.**
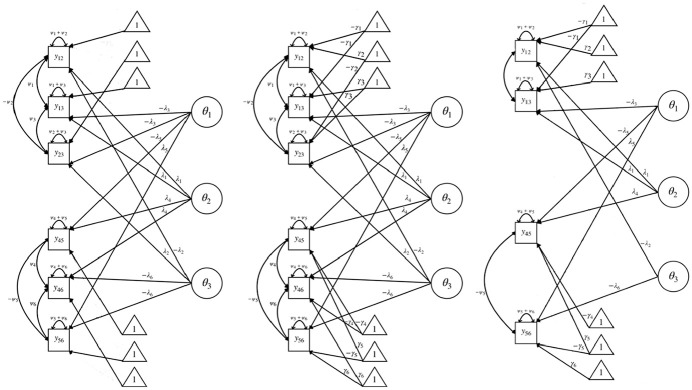
Path diagrams for the original T-IRT model (left), the version considering only stochastic dependencies (middle) and the version considering both stochastic and logical dependencies (left), for the answer pattern (2,1,3,1,2,3) . Correlations between θ are not displayed in the path diagrams for the sake of simplicity as they are identical for all possible T-IRT path diagrams.

To illustrate the theoretical implications of considering stochastic and logical dependencies, three path diagrams consisting of two blocks of length 3 are displayed in Figure 1. The left diagram shows the original T-IRT model, the one in the middle shows the version considering only stochastic dependencies and the right one shows the version considering both stochastic and logical dependencies, exemplary for the realized pattern (2,1,3,1,2,3) . Apart from the statement intercepts, all three diagrams include the same parameters. They only differ in the amount of included equations. The two diagrams considering stochastic dependencies include the same parameters and are equivalent.

## Simulation Study

To compare the performance of these three T-IRT implementations, *original*, *stochastic dependencies*, and *stochastic & logical dependencies*, a simulation study is performed. To gain insight into the performance depending on the conditions specified by the test constructor, we vary the block length, the test length, and the sample size in two settings each. The block length $n_{b}$ is of particular interest because it controls the strength of the logical dependencies. Since dependencies only occur for block lengths greater than 2 and the cognitive load increases with block length (Brown & Maydeu-Olivares, 2011), we investigate block lengths 3 and 4 . To vary the test lengths, the length of the resulting full binary patterns is varied between 30 (*short* test) and 60 (*medium* test). For the different block lengths, this means that K∈{10,20} blocks for $n_{b}=3$ and K∈{5,10} blocks for $n_{b}=4$ are investigated. The sample size is chosen to be J∈{50,200} . These factors are evaluated in a fully crossed design. Each of the 12 settings is evaluated with 50 replications. To keep the number of varied parameters to a minimum, we chose to keep factors that do not affect the dependencies between items constant. Such factors are the correlation between traits and the proportion of negatively keyed items. For these parameters, we tried to mimic values commonly found in practice. The response model is the T-IRT model with $\mu \sim^{\mathrm{iid}}U[-1,1]$ , $\lambda^{\left(1\right)}\sim^{\mathrm{iid}}U[0.65,0.95]$ , $\psi_{\mathrm{ii}}=1-{\left(\lambda_{i}^{\left(1\right)}\right)}^{2}$ , and θ~N(0,Σ) . The simulated tests measure five traits with a trait correlation Σ mimicking the “Big 5” from van der Linden et al. (2010). The proportion of unequally keyed items is kept at two-thirds. The models are implemented with weakly informative priors that should fit all common data. All parameters have a normally distributed prior with varying mean and standard deviation:

(14) $$\lambda_{i}\overset{iid}{\sim }N\left(1,0.5\right)\cdot 1_{\left[0,\infty \right]}\forall \lambda_{i}\in \lambda_{pos}$$

(15) $$\lambda_{i}\sim^{\mathrm{iid}}N\left(-1,\;0.5\right)\cdot 1_{\left(-\infty ,0\right]}\;\forall \lambda_{i}\in \lambda_{\mathrm{neg}},$$

(16) $$\mu_{i}\sim^{\mathrm{iid}}N\left(0,2\right)\;\forall i\in \{1,\ldots ,K\cdot \mathrm{nb}\},$$

(17) $$\psi_{i}\sim^{\mathrm{iid}}N\left(1,\;0.3\right)\cdot 1_{\left[0,\infty \right)}\;\forall i\in \{1,\ldots ,K\cdot \mathrm{nb}\},$$

(18) $$\theta_{j}\sim^{\mathrm{iid}}\mathrm{lkj}\;\mathrm{corr}\;\mathrm{cholesky}\left(1\right)\cdot N\left(0,1\right)\;\forall j\in \{1,\ldots ,J\},$$

with $\lambda_{\mathrm{pos}}$ being the set of factor loadings with positive item keying and $\lambda_{\mathrm{neg}}$ the ones with negative item keying. For drawing correlated trait parameters $\theta_{j}$ , the Cholesky LKJ correlation distribution, a common default prior for covariance and correlation matrices, is used. With the distribution’s scale parameter η being set to 1, the density over all correlation matrices is uniform. The intercept $\gamma_{\mathrm{il}}\;\forall i,l\in \{1,\ldots ,k\cdot n_{b},i<l\}$ results from the sum $\mu_{i}=\mu_{l}$ , as intercepts are drawn per item, this leads to γ~N(0,4) . According to Bürkner et al. (2019), these priors improve sampling efficiency and convergence while mildly influencing parameter estimates.

The simulation study and its evaluation are carried out using the software R (R Core Team, 2024) version 4.2.2. All implementations of the Thurstonian IRT model are implemented using Stan (Stan Development Team, 2024) software. The interface between R and Stan is established through the rstan package (Stan Development Team, 2022) version 2.26.1. The package mvtnorm (Genz et al., 2024) version 1.1-3 is used for data simulation,. The microbenchmark function from the package microbenchmark (Mersmann, 2024) version 1.4.10 was used to evaluate the computational time of each implementation. In addition, the package ggplot2 (Wickham, 2016) version 3.4.2 was used for visualization. The R-code for the simulation as well as the Stan-code of the three Thurstonian IRT model implementations are available on OSF https://osf.io/8fndw/.

## Simulation Results

**Figure 2.**
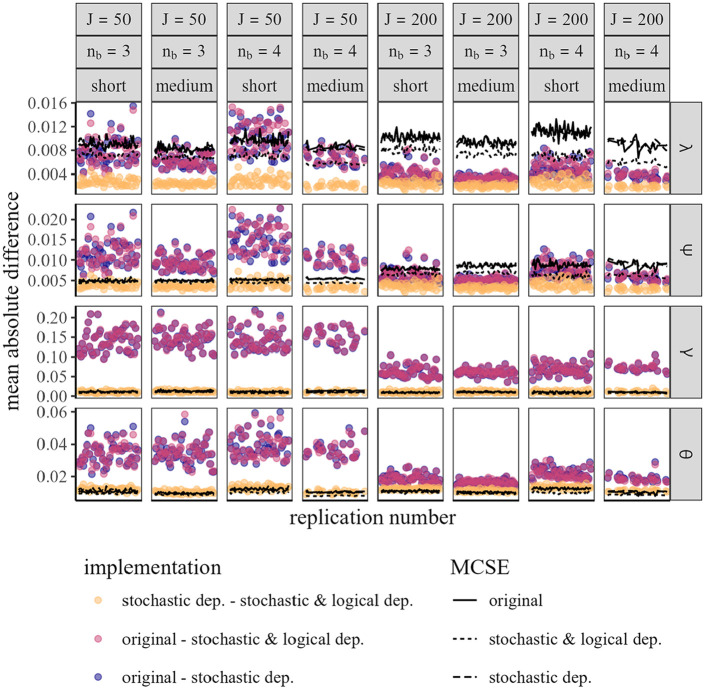
Average absolute difference between estimates of the estimation methods (dots) and the average MCSEs (lines) per setting and replication. *Note*. MCSE = Monte-Carlo standard error.

We compare deviations between estimates to evaluate the performance of the three implementations based on their parameter estimates. Figure 2 illustrates the differences between parameter estimates alongside the respective Monte-Carlo standard errors (MCSEs). It is visible that the parameters from the implementation *stochastic dep.* and *stochastic & logical dep.* share a higher similarity than the estimates resulting from the *original* implementation. This is expected behavior as we showed for block length 3 that these two implementations are equivalent. Differences between them are within or even below the MCSE. This strongly indicates that the equivalence holds true also for block lengths larger than 3. The intensity of difference between these two implementations and the *original* implementation depends on the simulation settings and the parameter group. For the factor loadings λ , differences between implementations are always within or below the MCSE. For the covariance of the errors Ψ , the differences are above the MCSE for the sample size 50 and within the sample size 200. For the intercepts γ and the latent traits θ , the difference is consistently above the standard error of the estimation. Since intercepts are defined differently in the implementations (constrained vs. unconstrained), these are expected to differ. This also affects the trait estimation, which is unexpected. However, the magnitude of those differences is small and therefore should have no practical relevance in use cases the authors envision.

Generally, differences between implementations decrease with increasing information, in the form of sample size and test length. When increasing the block length, the differences between the *original* implementation and the others increase. This behavior has two possible explanations. First, with increasing block length, dependencies increase, therefore ignoring them leads to a larger mismatch between the estimates. Second, since the number of full comparisons is held constant, the information contained in the test is smaller for larger block lengths, and therefore differences become larger.

The MCSE for the implementation *stochastic & logical dep.* is lower than for the other two implementations. This is an indicator that sampling is more efficient when considering stochastic and logical dependencies. This can be also seen when investigating convergence. While, according to the effective sample size and $\hat{R}$ , all models converged for all investigated samples, the estimated effective sample size was often a bit larger for the implementation *stochastic & logical dep.* rather than the other two implementations. This phenomenon has been exemplary illustrated in the empirical example in Table 2. Since there seems to be a systematic difference in efficiency per Monte-Carlo iteration, it is of interest to see whether this is reflected in computation time, too. Table 1 contains the average (and standard deviation) computation time over all 50 replications per setting. These times were achieved on a cluster running 4 parallel chains for each model for 2,000 iterations, using 4 cores per model.

**Table 1.** Average Run Times in Hours and Their Standard Deviations Over 50 Replications per Simulation Setting.

$n_{b}$	J	Test length	Considering logical and stochastic dependencies	Considering stochastic dependencies	Original
3	50	Short	0.23 (0.02)	2.43 (0.20)	2.64 (0.18)
3	50	Medium	0.81 (0.07)	9.14 (0.62)	9.75 (0.70)
3	200	Short	1.00 (0.07)	11.33 (0.75)	11.55 (0.88)
3	200	Medium	3.74 (0.40)	40.26 (2.91)	41.06 (2.90)
4	50	Short	0.16 (0.01)	2.59 (0.19)	2.75 (0.19)
4	50	Medium	0.49 (0.03)	9.17 (0.28)	9.68 (0.33)
4	200	Short	0.70 (0.05)	11.73 (0.89)	12.26 (1.11)
4	200	Medium	2.20 (0.12)	43.06 (2.77)	46.37 (4.56)

It is obvious that across settings, the required computation time is at least by the factor 10 smaller in the implementation *stochastic & logical dep.* than in the other two implementations. The difference between those is way smaller; however, the implementation *stochastic dep.* is always a bit faster than the *original* implementation. In addition, it is interesting to see that the ratio between implementations is stable for both sample sizes. This indicates that the larger the sample gets the greater the added value of the implementation *stochastic & logical dep.* Unsurprisingly, the longer the test, the higher the required computation time.

The computation time changes only mildly with the block length. However, an interesting phenomenon is observable. While computation time mildly increases with increasing block length for the two implementations *stochastic dep.* and *original*, it decreases for the implementation *stochastic & logical dep.* This behavior can be explained with the simulation setup. A larger block length leads to more dependencies between the statement comparisons if all binary comparisons are considered, this increases the computation time for those implementations. However, since the number of all binary compared statements is held constant, the number of independent comparisons decreases with block length resulting in a faster computation time for the implementation *stochastic & logical dep*.

## Empirical Example

To demonstrate that all three versions of the Thurstonian IRT model implementation can be fitted to real-world data and to explore the practical implications of these implementations, the models were applied to data from a personality test. The test is a modified version of the Big Five Inventory-2 (BFI-2) in a forced-choice design, measuring the five personality traits *openness*, *conscientiousness*, *extraversion*, *agreeableness*, and *neuroticism*. Each of these traits is assessed with 12 statements, resulting in a total of 60 statements. The statements are presented in pairs of 3, resulting in 20 blocks. Each block consists of positively and negatively coded statements. The proportion of positively and negatively coded items is almost balanced, with 29 positively and 31 negatively coded statements. The test was administered to 1,031 participants, of whom 94 were excluded from the analysis due to missing values. The data were collected in the study by Kupffer et al. (2024). In this study, participants were asked to complete six questionnaires, one of which was the BFI-2. The data were collected in an online survey that was conducted over a 2-week period in September and October 2017. All participants were from English-speaking countries, with a mean age of 36 years (*SD* = 11), and 46% of the participants were male. More details about the data collection process and the test can be found in the original study documentation from Kupffer et al. (2024).

All the implementations of the Thurstonian IRT model converged when fitting them to the data. This can be seen in Table 2, as all $\hat{R}$ values fall below the threshold of 1.1 and nearly all parameters have an effective sample size beyond 400. It is to see that $\hat{R}$ and $n_{\mathrm{eff}}$ indicate better convergence for the implementation *stochastic & logical dep.* rather than the two competing implementations. This is also supported by differences in computation time. While the implementation *stochastic & logical dep.* already took 14 hr to run, *stochastic dep.* and *original* took 99 hr.

**Table 2.** Effective Sample Size ( $n_{\mathrm{eff}}$ ) and $\hat{R}$ in the Empirical Example per Implementation.

implementation	Mean $\hat{R}$	Max $\hat{R}$	Mean $n_{\mathrm{eff}}$	Min $n_{\mathrm{eff}}$
Original	1.00	1.02	7,146.31	360.65
Stochastic & logical dep.	1.00	1.01	8,413.24	818.42
Stochastic dep.	1.00	1.02	6,109.67	381.73

**Figure 3.**
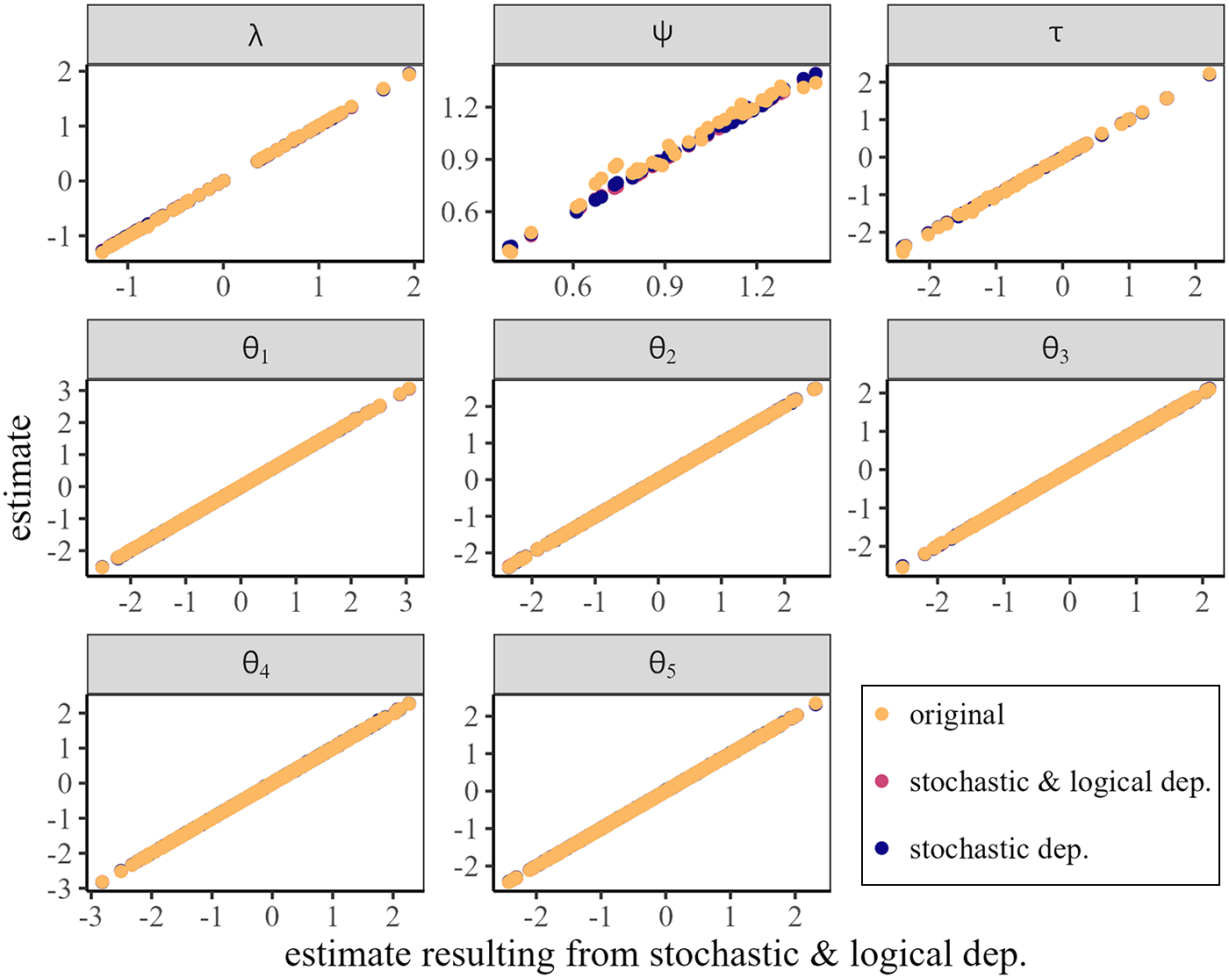
Scatterplot of estimated model parameters for each implementation plotted against those resulting from the implementation *stochastic & logical dep.*

When investigating the point estimates one can see, analogous to the simulation results, a high agreement between the parameter estimates of all three implementations, see Figure 3. One can see that the estimates of all models align nearly perfectly, which shows that the parameter recovery is not strongly affected by the choice of implementation.

To see whether the small differences affect the model fit, we compare the widely applicable information criterion (WAIC) for the three models. As illustrated in Table 3, the implementation *stochastic & logical dependencies* should be preferred, as it resulted in the highest predictive accuracy. Followed by the implementation *stochastic dependencies* and *original* having the lowest predictive accuracy. However, part of the difference in fit is due to the difference in the constants of the multivariate normal distributions. As we note in Equation (A7) in the Appendix, the general density of a singular normal distribution is $\frac{{\left(2\pi \right)}^{-r/2}}{\sqrt{v_{1}\ldots v_{r}}}$ , where $v_{1},\ldots v_{r}$ are the non-zero eigenvalues. Consequently, a three-dimensional multivariate normal with factor ${\left(2\pi \right)}^{-3/2}$ differs from the correct factor of a two-dimensional multivariate normal by a factor of $1/\sqrt{2\pi }.$ Each multivariate observation hence changes the log-likelihoods by $\ln\left(1/\sqrt{2\pi }\right)\approx -0.92$ , which sums roughly to the magnitude of the observed differences in elpds. In addition, small differences can stem from differences in convergence and the Markov chain Monte Carlo (MCMC) sampling. Overall, the fit appears to increase slightly when using the implementation *stochastic & logical dependencies*. Although this only leads to small differences in the parameter estimates, the substantial reduction in computation time might be of interest to practitioners.

**Table 3.** Estimated WAIC Values in the Empirical Example per Implementation.

implementation	elpd_waic	se_elpd_waic	elpd_diff	se_diff
Stochastic & logical dependencies	−74,494.78	77.43	0.00	0.00
Stochastic dependencies	−74,967.73	80.62	−472.95	19.57
Original	−75,154.02	79.86	−659.24	19.34

*Note*. WAIC = widely applicable information criterion.

## Discussion

The objective of this study is to investigate whether the parameter estimates of the Bayesian Thurstonian IRT model are affected by the consideration of dependencies within blocks. In the originally defined Thurstonian IRT model, two types of dependencies occur. One are stochastic dependencies, resulting in illogical answer patterns, which can be avoided by constraining the utility intercepts. The other are logical dependencies at the test taker level due to redundant information in binary comparisons. These can be eliminated by considering only item comparisons with neighboring ranks. A theoretical comparison was made between the likelihoods of implementations that consider and neglect logical dependencies on the test taker level while considering stochastic dependencies. The comparison showed that for a block length of 3, the likelihoods are identical. Since both implementations are based on the same item and trait parameters with identical prior distribution, this results in equal posterior estimates. The authors assume that the proof generalizes to block lengths larger than 3.

To investigate the effect of constrained intercepts on parameter estimation, a simulation study was conducted. The study showed that accounting for stochastic dependencies leads to estimates that are as accurate, if not slightly more accurate, than those from the original T-IRT model. Since constraining the model has only been proposed by Brown and Maydeu-Olivares (2011) to enable person parameter estimation, there is no theoretical reasoning not to constrain the intercepts. Furthermore, by constraining the intercepts, the model adheres to Thurstone’s (1927) Law of Comparative Judgment. As such, it reflects the ranking process of individuals more accurately, as they cannot give intransitive rankings. Therefore, since current software enables us to consider stochastic dependencies, these should be considered in the Thurstonian IRT model.

The two implementations that consider stochastic dependencies but either consider or neglect logical dependencies are highly similar for all investigated settings. Especially interesting is that this similarity did not change with block lengths (3 and 4). While this supports the assumption of equivalent parameter estimates, the computational efficiency in the forms of convergence and computation time differs strongly between these implementations. When additional logical dependencies were considered, the computation time decreased drastically alongside a decrease in the MCSEs.

Therefore, considering both stochastic and logical dependencies in Thurstonian IRT model estimation has several advantages without any drawbacks. We recommended users utilize a T-IRT implementation that considers logical dependencies. For those interested in Bayesian model estimation, this paper provides the necessary code. The provided Bayesian implementation has the huge advantage that it can be easily extended. Researchers interested in extensions like multi-group models can adapt the stan code by changing only a few lines. Nevertheless, this idea can also be employed for frequentist estimation. Future research could implement this idea in frequentist models, to enable an even faster implementation.

## Appendix

### Equivalence of Estimation With and Without Logical Dependencies

To show the equivalence between the estimation with and without logical dependencies. For this, the equality of both densities for block length $n_{b}=3$ is shown in the following. For block lengths larger than 3, the idea of the proof remains the same. In the following, the focus is on the covariance matrix. Without loss of generality, it is assumed that the multivariate mean α=−γ+Λθ are zero. The case for other mean vectors can be easily obtained by adding α to the random variable. The intercepts for both approaches are equal because they depend on the same parameters which are identical for both approaches. As observations between blocks are independent conditional on θ , for the sake of simplicity in the following one block of statements is discussed, so the block diagonal matrix Ψ consists of one block only.

Let us assume $y_{i}^{*\mathrm{new}}\sim N\left(0,{\tilde{\Psi }}_{i}^{\mathrm{new}}\right)$ , i=1,2,3 are the random variables of utility differences if there are no logical dependencies in the data and $y^{*}\sim N\left(0,\tilde{\Psi }\right)$ are the random variables if all binary comparisons are considered. Then, $y_{i}^{*\mathrm{new}}\in R^{n_{b}-1}$ is a sub-vector of $y^{*}\in R^{n_{b}\left(n_{b}-1\right)/2}$ with:

(A1)
$$\begin{array}{c} {\tilde{\Psi }}_{1}^{\mathrm{new}}=\left(\begin{array}{cc} \psi_{1}+\psi_{2} & \psi_{1}\\ \psi_{1} & \psi_{1}+\psi_{3} \end{array}\right),{\tilde{\Psi }}_{2}^{\mathrm{new}}=\left(\begin{array}{cc} \psi_{1}+\psi_{2} & -\psi_{2}\\ -\psi_{2} & \psi_{2}+\psi_{3} \end{array}\right)\mathrm{and}\;\\ {\tilde{\Psi }}_{3}^{\mathrm{new}}=\left(\begin{array}{cc} \psi_{1}+\psi_{3} & \psi_{3}\\ \psi_{3} & \psi_{2}+\psi_{3} \end{array}\right),\mathrm{and} \end{array}$$

(A2)
$$\tilde{\Psi }=\left(\begin{array}{ccc} \psi_{1}+\psi_{2} & \psi_{1} & -\psi_{2}\\ \psi_{1} & \psi_{1}+\psi_{3} & \psi_{3}\\ -\psi_{2} & \psi_{3} & \psi_{2}+\psi_{3} \end{array}\right),$$

with $\mathrm{rank}\left(\tilde{\Psi }\right)=r=2<3$ and $\mathrm{rank}\left({\tilde{\Psi }}_{i}^{\mathrm{new}}\right)=r=2,\;i=1,\;2,\;3$ depending on the test takers’ response pattern. In the following, only $\tilde{\Psi }$ and ${\tilde{\Psi }}_{1}^{\mathrm{new}}$ are considered as the proof is parallel for all i . The utility differences are $y^{*}={\left(y_{1}^{*},y_{2}^{*},y_{3}^{*}\right)}^{'}$ , and setting $w_{1}=\left(\begin{array}{ccc} 1 & 0 & 0\\ 0 & 1 & 0 \end{array}\right)$ , the product $w_{1}y^{*}=y_{1}^{*\mathrm{new}}$ has covariance ${\tilde{\Psi }}_{1}^{\mathrm{new}}$ .

The inverse square root of the determinant of ${\tilde{\Psi }}_{i}^{\mathrm{new}}$ is ${\left|{\tilde{\Psi }}_{i}^{\mathrm{new}}\right|}^{-1/2}=\frac{1}{\sqrt{\psi_{1}\psi_{2}+\psi_{1}\psi_{3}+\psi_{2}\psi_{3}}}$ , for i=1,2,3 . The inverse of ${\tilde{\Psi }}_{i}^{\mathrm{new}}$ depends on i . For i=1 , it is given by:

(A3)
$$\begin{array}{c} {\left({\tilde{\Psi }}_{1}^{\mathrm{new}}\right)}^{-1}=\frac{1}{\psi_{1}\psi_{2}+\psi_{1}\psi_{3}+\psi_{2}\psi_{3}}\left(\begin{array}{cc} \psi_{1}+\psi_{3} & -\psi_{1}\\ -\psi_{1} & \psi_{1}+\psi_{2} \end{array}\right). \end{array}$$

Plugging this information into the density distribution of a regular bivariate normal distribution results in:

(A4)
$$g\left(y_{1}^{*\mathrm{new}}\right)=\;g\left(w_{1}\;y^{*}\right)=\;g\left(\left(\begin{array}{c} y_{1}^{*}\\ y_{2}^{*} \end{array}\right)\right)\;=\;{\left(2\pi \right)}^{-r/2}\;{\left|{\tilde{\Psi }}_{1}^{\mathrm{new}}\right|}^{-1/2}\exp\left(-\;\frac{1}{2}\;{\left(y_{1}^{*\mathrm{new}}\right)}^{T}{\left({\tilde{\Psi }}_{1}^{\mathrm{new}}\right)}^{-1}y_{1}^{*\mathrm{new}}\right)$$

(A5)
$$\begin{array}{c} \;\;\;\;\;\;\;\;=\;\frac{1}{2\pi }\;\frac{1}{\sqrt{\psi_{1}\psi_{2}+\psi_{1}\psi_{3}+\psi_{2}\psi_{3}}}\\ \;\;\;\;\;\;\;\exp\left(-\;\frac{1}{2}{\left(\begin{array}{c} y_{1}^{*}\\ y_{2}^{*} \end{array}\right)}^{T}\frac{1}{\psi_{1}\psi_{2}+\psi_{1}\psi_{3}+\psi_{2}\psi_{3}}\left(\begin{array}{cc} \psi_{1}+\psi_{3} & -\psi_{1}\\ -\psi_{1} & \psi_{1}+\psi_{2} \end{array}\right)\left(\begin{array}{c} y_{1}^{*}\\ y_{2}^{*} \end{array}\right)\right) \end{array}$$

(A6)
$$\;\;\;\;\;\;\;\;\;=\;\frac{1}{2\pi }\;\frac{1}{\sqrt{\psi_{1}\psi_{2}+\psi_{1}\psi_{3}+\psi_{2}\psi_{3}}}\exp\left(\frac{\left(\left(\psi_{1}+\psi_{3}\right)y_{1}^{2}-2\psi_{1}y_{1}y_{2}+\left(\psi_{1}+\psi_{2}\right)y_{2}^{2}\right)}{-2\psi_{1}\psi_{2}+\psi_{1}\psi_{3}+\psi_{2}\psi_{3}}\right).$$

As $\tilde{\Psi }$ does not have full rank $y^{*}$ , stems from a singular multivariate normal distribution. The general density of the singular normal distribution is defined as:

(A7)
$$f\left(y^{*}\right)={\left(2\pi \right)}^{-r/2}\frac{1}{\sqrt{\left(\upsilon_{1}\cdot \ldots \cdot \upsilon_{r}\right)}}\exp\left(-\frac{1}{2}{\left(y^{*}\right)}^{T}{\tilde{\Psi }}^{-}y^{*}\right)$$

with ${\tilde{\Psi }}^{-}$ the *g*-inverse of $\tilde{\Psi }$ and $\upsilon_{1},\ldots ,\upsilon_{r}$ the nonzero eigenvalues of $\tilde{\Psi }$ (Härdle & Simar, 2019). The eigenvalues can be derived by solving $\left|\tilde{\Psi }-\mathrm{diag}\left(\lambda \right)\right|\overset{!}{=}0$

(A8)
$$\left|\tilde{\Psi }-\mathrm{diag}\left(\lambda \right)\right|=\left|\begin{array}{ccc} \psi_{1}+\psi_{2}-\lambda & \psi_{1} & -\psi_{2}\\ \psi_{1} & \psi_{1}+\psi_{3}-\lambda & \psi_{3}\\ -\psi_{2} & \psi_{3} & \psi_{2}+\psi_{3}-\lambda \end{array}\right|$$

(A9)
$$\begin{array}{c} =\left(\psi_{1}+\psi_{2}-\lambda \right)\left(\psi_{1}+\psi_{3}-\lambda \right)\left(\psi_{2}+\psi_{3}-\lambda \right)-\left(\psi_{1}\psi_{2}\psi_{3}\right)-\left(\psi_{1}\psi_{2}\psi_{3}\right)\\ -\psi_{2}^{2}\left(\psi_{1}+\psi_{3}\right)-\psi_{3}^{2}\left(\psi_{1}+\psi_{2}\right)-\psi_{1}^{2}\left(\psi_{2}+\psi_{3}\right) \end{array}$$

(A10)
$$\begin{array}{c} =\left(\psi_{1}^{2}+\psi_{1}\psi_{3}-\psi_{1}\lambda +\psi_{2}\psi_{1}+\psi_{2}\psi_{3}-\psi_{2}\lambda -\psi_{1}\lambda -\psi_{3}\lambda +\lambda^{2}\right)\left(\psi_{2}+\psi_{3}-\lambda \right)\\ \;-\left(\psi_{1}\psi_{2}\psi_{3}\right)-\left(\psi_{1}\psi_{2}\psi_{3}\right)-\psi_{2}^{2}\left(\psi_{1}+\psi_{3}\right)-\psi_{3}^{2}\left(\psi_{1}+\psi_{2}\right)-\psi_{1}^{2}\left(\psi_{2}+\psi_{3}\right) \end{array}$$

(A11)
$$\begin{array}{c} =\psi_{1}^{2}\psi_{2}+\psi_{1}\psi_{2}\psi_{3}-\psi_{1}\psi_{2}\lambda +\psi_{1}\psi_{2}^{2}+\psi_{2}^{2}\psi_{3}-\psi_{2}^{2}\lambda -\psi_{1}\psi_{2}\lambda -\psi_{2}\psi_{3}\lambda +\psi_{2}\lambda^{2}\\ \;+\psi_{1}^{2}\psi_{3}+\psi_{1}\psi_{3}^{2}-\psi_{1}\psi_{3}\lambda +\psi_{1}\psi_{2}\psi_{3}+\psi_{2}\psi_{3}^{2}-\psi_{2}\psi_{3}\lambda -\psi_{1}\psi_{3}\lambda -\psi_{3}^{2}\lambda +\psi_{3}\lambda^{2}\\ \;-\psi_{1}^{2}\lambda -\psi_{1}\psi_{3}\lambda +\psi_{1}\lambda^{2}-\psi_{1}\psi_{2}\lambda -\psi_{2}\psi_{3}\lambda +\psi_{2}\lambda^{2}+\psi_{1}\lambda^{2}+\psi_{3}\lambda^{2}-\lambda^{3}-\psi_{1}\psi_{2}\psi_{3}\\ \;-\psi_{1}\psi_{2}\psi_{3}-\psi_{2}^{2}\psi_{1}-\psi_{2}^{2}\psi_{3}+\psi_{2}^{2}\lambda -\psi_{3}^{2}\psi_{1}-\psi_{2}\psi_{3}^{2}+\psi_{3}^{2}\lambda -\psi_{1}^{2}\psi_{2}-\psi_{1}^{2}\psi_{3}+{\lambda \psi }_{1}^{2} \end{array}$$

(A12)
$$=\lambda \left(3\left(-\psi_{1}\psi_{2}-\psi_{1}\psi_{3}-\psi_{2}\psi_{3}\right)+2\lambda \left(\psi_{1}+\psi_{2}+\psi_{3}\right)-\lambda^{2}\right)$$

The solutions to $\left|\tilde{\Psi }-\mathrm{diag}\left(\lambda \right)\right|{=}^{!}0$ are than

(A13)
$$\mathrm{case}\;1:\;\lambda_{2}=0,$$

(A14)
$$\mathrm{case}\;2:\;\lambda_{2}=\psi_{1}+\psi_{2}+\psi_{3}+\sqrt{{\left(\psi_{1}+\psi_{2}+\psi_{3}\right)}^{2}-3\left(\psi_{1}\psi_{2}+\psi_{1}\psi_{3}+\psi_{2}\psi_{3}\right)}\;\mathrm{and}$$

(A15)
$$\mathrm{case}\;3:\;\lambda_{3}=\psi_{1}+\psi_{2}+\psi_{3}-\sqrt{{\left(\psi_{1}+\psi_{2}+\psi_{3}\right)}^{2}-3\left(\psi_{1}\psi_{2}+\psi_{1}\psi_{3}+\psi_{2}\psi_{3}\right)}.$$

with cases 2 and 3 resulting from the quadratic formula. The product of the non-zero eigenvalues of $\tilde{\Psi }$ is:

(A16)
$$\lambda_{2}\cdot \lambda_{3}=\psi_{1}^{2}+\psi_{1}\psi_{2}+\psi_{1}\psi_{3}+\psi_{1}\psi_{2}+\psi_{2}^{2}+\psi_{2}\psi_{3}+\psi_{1}\psi_{3}+\psi_{2}\psi_{3}+\psi_{3}^{2}$$

(A17)
$$-{\left(\sqrt{{\left(\psi_{1}+\psi_{2}+\psi_{3}\right)}^{2}-3\left(\psi_{1}\psi_{2}-\psi_{1}\psi_{3}-\psi_{2}\psi_{3}\right)}\right)}^{2}$$

(A18)
$$=3\left(\psi_{1}\psi_{2}+\psi_{1}\psi_{3}+\psi_{2}\psi_{3}\right).$$

To obtain the g-inverse of $\tilde{\Psi }$ , the matrix can be partitioned into four sub-matrices:

(A19)
$$\begin{array}{c} \tilde{\Psi }=\left(\begin{array}{cc} P_{11} & P_{12}\\ P_{21} & P_{22} \end{array}\right),\;\mathrm{with}\;P_{11}=\left(\begin{array}{cc} \psi_{1}+\psi_{2} & \psi_{1}\\ \psi_{1} & \psi_{1}+\psi_{3} \end{array}\right),\;P_{12}=\left(\begin{array}{c} -\psi_{2}\\ \psi_{3} \end{array}\right),\\ P_{21}=\left(\begin{array}{cc} -\psi_{2} & \psi_{3} \end{array}\right)\;\mathrm{and}\;P_{22}=\left(\psi_{2}+\psi_{3}\right) \end{array}$$

with $P_{11}$ being a non-singular matrix. Therefore ${\tilde{\Psi }}^{-}$ , the generalized inverse of $\tilde{\Psi }$ can be defined as:

(A20)
$${\tilde{\Psi }}^{-}=\left(\begin{array}{cc} P_{11}^{-1} & 0\\ 0 & 0 \end{array}\right)=\frac{1}{\psi_{1}\psi_{2}+\psi_{1}\psi_{3}+\psi_{2}\psi_{3}}\left(\begin{array}{ccc} \psi_{1}+\psi_{3} & -\psi_{1} & 0\\ -\psi_{1} & \psi_{1}+\psi_{2} & 0\\ 0 & 0 & 0 \end{array}\right).$$

Two alternative generalized inverses can be found with the use of $Z=U_{m}\tilde{\Psi }V_{m}$ , m=1,2 , with the permutation matrices $U_{1}=V_{1}=\left(\begin{array}{ccc} 1 & 0 & 0\\ 0 & 0 & 1\\ 0 & 1 & 0 \end{array}\right)$ and $V_{2}=U_{2}^{T}=\left(\begin{array}{ccc} 0 & 1 & 0\\ 0 & 0 & 1\\ 1 & 0 & 0 \end{array}\right)$ resulting in two alternative *g*-inverses:

(A21)
$$\begin{array}{c} \frac{1}{\psi_{1}\psi_{2}+\psi_{1}\psi_{3}+\psi_{2}\psi_{3}} \end{array}\left(\begin{array}{ccc} \psi_{2}+\psi_{3} & 0 & \psi_{2}\\ 0 & 0 & 0\\ \psi_{2} & 0 & \psi_{1}+\psi_{3} \end{array}\right)\;\mathrm{and}\frac{1}{\psi_{1}\psi_{2}+\psi_{1}\psi_{3}+\psi_{2}\psi_{3}}\left(\begin{array}{ccc} 0 & 0 & 0\\ 0 & \psi_{2}+\psi_{3} & -\psi_{3}\\ 0 & -\psi_{3} & \psi_{1}+\psi_{3} \end{array}\right).$$

Substituting any of these three generalized inverses in Equation (A7) results in identical densities of $y^{*}$ , all given by:

(A22)
$$\begin{array}{cc} f\left(y^{*}\right) & =\frac{1}{2\pi \sqrt{3\left(\psi_{1}\psi_{2}+\psi_{1}\psi_{3}+\psi_{2}\psi_{3}\right)}}\exp\left(\frac{\left(\psi_{1}+\psi_{3}\right){\left(y_{1}^{*}\right)}^{2}-2\psi_{1}y_{1}^{*}y_{2}^{*}+\left(\psi_{1}+\psi_{2}\right){\left(y_{2}^{*}\right)}^{2}}{-2\left(\psi_{1}\psi_{2}+\psi_{1}\psi_{3}+\psi_{2}\psi_{3}\right)}\right) \end{array}.$$

That this density of $y^{*}$ only depends on the value of two arbitrary utility differences can be explained by the linear relationship $y_{1}^{*}=y_{2}^{*}-y_{3}^{*}$ . With the knowledge of two differences, the third one can be directly calculated. Hence, the density does not depend on which pairs are considered. Implicitly in those three expressions, a Dirac delta function is included which is zero if this relationship is not fulfilled.

We consider the likelihood of test taker j and assume the binary code of the answer pattern is $\left(b_{1},b_{2},b_{3}\right)=\left(1,\;0,\;0\right)$ or (0,1,1) . Then, the utility differences modeled when considering dependencies are $y_{1}^{*}$ and $y_{2}^{*}$ . Finally, we show the densities are identical:

(A23)
$$P\left(y=b|\theta_{j},\gamma ,\Lambda ,\tilde{\Psi }\right)$$

(A24)
$$=\;\int_{-\infty }^{\infty }\int_{-\infty }^{\infty }\int_{-\infty }^{\infty }f(y^{*})\;\cdot \delta_{y_{3}^{*}}\;(y_{2}^{*}-\;y_{1}^{*})\cdot \overset{3}{\underset{i=1}{\Pi }}u_{y_{i}^{*}}\;(b_{i})dy^{*}$$

(A25)
$$\begin{array}{c} =\int_{-\infty }^{\infty }\int_{-\infty }^{\infty }\int_{-\infty }^{\infty }\frac{1}{2\pi \sqrt{3\left(\psi_{1}\psi_{2}+\psi_{1}\psi_{3}+\psi_{2}\psi_{3}\right)}}\\ \exp\left(\frac{\left(\left(\psi_{1}+\psi_{3}\right){\left(y_{1}^{*}\right)}^{2}-2\psi_{1}y_{1}^{*}y_{2}^{*}+\left(\psi_{1}+\psi_{2}\right){\left(y_{2}^{*}\right)}^{2}\right)}{-2\left(\psi_{1}\psi_{2}+\psi_{1}\psi_{3}+\psi_{2}\psi_{3}\right)}\right)\;\cdot \delta_{y_{3}^{*}}\;(y_{2}^{*}-\;y_{1}^{*})\cdot \overset{3}{\underset{i=1}{\Pi }}u_{y_{i}^{*}}\;(b_{i})dy^{*} \end{array}$$

(A26)
$$\begin{array}{c} \overset{\left(**\right)}{=}\int_{-\infty }^{\infty }\int_{-\infty }^{\infty }\frac{1}{2\pi \sqrt{3\left(\psi_{1}\psi_{2}+\psi_{1}\psi_{3}+\psi_{2}\psi_{3}\right)}}\\ \exp\left(\frac{\left(\left(\psi_{1}+\psi_{3}\right){\left(y_{1}^{*}\right)}^{2}-2\psi_{1}y_{1}^{*}y_{2}^{*}+\left(\psi_{1}+\psi_{2}\right){\left(y_{2}^{*}\right)}^{2}\right)}{-2\left(\psi_{1}\psi_{2}+\psi_{1}\psi_{3}+\psi_{2}\psi_{3}\right)}\right)\cdot \overset{2}{\underset{i=1}{\Pi }}u_{y_{i}^{*}}\;\left(b_{i}\right)\sqrt{3}{\mathrm{dy}}_{1}^{*}{\mathrm{dy}}_{2}^{*} \end{array}$$

(A27)
$$\int_{-\infty }^{\infty }\int_{-\infty }^{\infty }g\left(w_{1}y^{*}\right)\cdot \overset{2}{\underset{i=1}{\Pi }}u_{y_{i}^{*}}\;\left(b_{i}\right)d\left(w_{1}y^{*}\right)$$

(A28)
$$P\left(y_{1}=b_{1},\;y_{2}=b_{2}\;|\theta_{j},\gamma ,\Lambda ,{\tilde{\Psi }}^{\mathrm{new}}\right),$$

with the Dirac delta function $\delta_{y_{3}^{*}}\left(y_{1}^{*}-y_{2}^{*}\right)$ and $u_{y_{i}^{*}}\left(b_{i}\right)=\left\{ \begin{array}{c} 1_{\left[0,\infty \right)}\left(y_{i}^{*}\right)\;b_{i}=1\\ 1_{\left(-\infty ,0\right]}\left(y_{i}^{*}\right)\;b_{i}=0 \end{array}\right.$ shows that the likelihoods for the approach neglecting logical dependence are identical with the likelihood of the approach considering logical dependencies. Note that for the equation marked with (**), we have used the change of variables formula, that is, the integral of the function h:U→R with respect to the transformation γ:Ω→U is given by $\int_{U}h\left(s\right)\mathrm{ds}:=\int_{\Omega }h\left(\gamma \left(v\right)\right)\cdot \sqrt{g^{\gamma }\left(v\right)}\mathrm{dv}$ . Here, $U\subset R^{3}$ , $\Omega \subset R^{2}$ , with the functions h:U→R , $h\left(\begin{array}{c} y_{1}\\ y_{2}\\ y_{3} \end{array}\right)=f\left(y^{*}\right)\cdot \delta_{y_{3}^{*}}\left(y_{2}^{*}-y_{1}^{*}\right)$ and $\gamma :\Omega \to U;\gamma \left(v\right)=\left(\begin{array}{cc} 1 & 0\\ 0 & 1\\ -1 & 1 \end{array}\right)\left(\begin{array}{c} y_{1}\\ y_{2} \end{array}\right)=\left(\begin{array}{c} y_{1}\\ y_{2}\\ y_{2}-y_{1} \end{array}\right)$ and the determinant

(A29)
$$g^{\gamma }\left(u\right)=\det\left(\left(\begin{array}{ccc} 1 & 0 & -1\\ 0 & 1 & 1 \end{array}\right)\left(\begin{array}{cc} 1 & 0\\ 0 & 1\\ -1 & 1 \end{array}\right)\right)=\det\left(\begin{array}{cc} 2 & -1\\ -1 & 2 \end{array}\right)=3.$$

In sum, the model’s likelihood depends on the same parameters and since the same priors are used regardless of whether logical dependencies occur, the posterior density is the same for both cases.
